# Dynamic Morphological Changes Induced By GM1 and Protein Interactions on the Surface of Cell-Sized Liposomes

**DOI:** 10.3390/ma6062522

**Published:** 2013-06-19

**Authors:** Shruti Dhingra, Masamune Morita, Tsuyoshi Yoda, Mun’delanji C. Vestergaard, Tsutomu Hamada, Masahiro Takagi

**Affiliations:** School of Material Science, Japan Advanced Institute of Science and Technology, Asahidai Nomi Ishikawa 923-1292, Japan; E-Mails: shruti.dhingra1988@gmail.com (S.D.); m-morita@jaist.ac.jp (M.M.); yoda.tsuyoshi@jaist.ac.jp (T.Y.); munde@jaist.ac.jp (M.C.V.); t-hamada@jaist.ac.jp (T.H.)

**Keywords:** cell membrane dynamics, liposome, surface interactions, ganglioside GM1, cholera toxin B (Ctb)

## Abstract

It is important to understand the physicochemical mechanisms that are responsible for the morphological changes in the cell membrane in the presence of various stimuli such as osmotic pressure. Lipid rafts are believed to play a crucial role in various cellular processes. It is well established that Ctb (Cholera toxin B subunit) recognizes and binds to GM1 (monosialotetrahexosylganglioside) on the cell surface with high specificity and affinity. Taking advantage of Ctb-GM1 interaction, we examined how Ctb and GM1 molecules affect the dynamic movement of liposomes. GM1 a natural ligand for cholera toxin, was incorporated into liposome and the interaction between fluorescent Ctb and the liposome was analyzed. The interaction plays an important role in determining the various surface interaction phenomena. Incorporation of GM1 into membrane leads to an increase of the line tension leading to either rupture of liposome membrane or change in the morphology of the membrane. This change in morphology was found to be GM1 concentration specific. The interaction between Ctb-GM1 leads to fast and easy rupture or to morphological changes of the liposome. The interactions of Ctb and the glycosyl chain are believed to affect the surface and the curvature of the membrane. Thus, the results are highly beneficial in the study of signal transduction processes.

## 1. Introduction

Regulation of lipid membrane dynamics is critical for many cellular processes [1]. It is important to understand the physicochemical mechanism that governs the morphological changes or responses in a cell membrane structure to various external and internal stimuli. Hence we used cell-sized (>10 µm) liposomes that mimic the natural cell structure to study the membrane dynamics. The detailed study of membrane dynamics in the presence of gangliosides can be helpful in understanding the mechanism of membrane dynamics of actual living cells [2]. Here we prepared liposomes containing GM1, monosialotetrahexosylganglioside, which is a component of raft domains in cell membrane and has an important role in signal transduction [3]. Rafts are dynamic clusters composed largely of cholesterol and sphingolipids, which are rich in highly ordered saturated acyl chains [4]. In this decade, the formation of lipid micro domain in mammalian plasma membrane, so called lipid raft, has attracted a lot of interest since lipid rafts are assumed to have functions as platforms of membrane associated events such as signal transduction, cell adhesion, lipid/protein sorting [5]. Although the steady state existence, size and shape of lipid rafts (liquid ordered micro domains) in plasma membrane still remain the subject of debate, lipid rafts can be considered essentially as a dynamic assembly of a variety of lipids and proteins. As knowledge of lipid raft functions in cellular signaling has been accumulated, the present agreement has been reached on the fact that raft domains coalesce upon cross-linking to form signaling and sorting platforms [6]. A common feature of lipid rafts is their peculiar lipid composition, being rich in glycosphingolipids (GSLs), sphingomyelin and cholesterol. Functions of lipid rafts are assumed to relate closely to the peculiar features of GSL molecules both in ceramide and oligosaccharide portions that can form complex hydrogen bonding networks (hydrogen bond donor and acceptor) [7]. GM1 refers to the GM1 ganglioside, one of the glycosphingolipids widely distributed in all tissues, but occurring in highest concentrations in the central nervous system. It is primarily located in the outer surface of the mammalian cells plasma membrane and in synaptic membranes of the CNS. GM1 modulates a number of cell surface and receptor activities as well as neuronal differentiation and development, protein phosphorylation and synaptic function [8]. GM1 is found embedded in the membrane of most cells. At one end, it has two long and hydrophobic acyl chains that anchor into the membrane providing cellular binding sites for proteins such as Ctb. It has been relatively easy to formulate liposomes containing ganglioside [9].

In our proposed model, GM1 clusters in homogeneous liposome in the presence of 1 mM osmotic pressure lead to the formation of small sphero-stomatocyte along with other transformations.

## 2. Results

In this study, we examined how constituent molecules affect the dynamic movement of liposomes. We used cell-sized lipid vesicles to enable direct observation of these changes using a phase contrast microscope. We observed the morphological changes and surface interactions taking place on the surface of homogeneous and raft exhibiting membranes under osmotic pressure. The following results indicate the various morphological changes observed on introduction of GM1 and Ctb on homogeneous and heterogeneous membranes. Interestingly there is an optimum concentration of GM1 at which separation of phases (or sphero-stomatocyte formation in case of homogeneous liposome) takes place. GM1 at a certain optimum concentration leads to restriction of diversity and leads to the formation of more stable endocytic vesicles (negative curvature). At this optimum concentration, the membrane might be saturated with GM1.

### 2.1. Homogeneous Liposome

**Figure 1:**
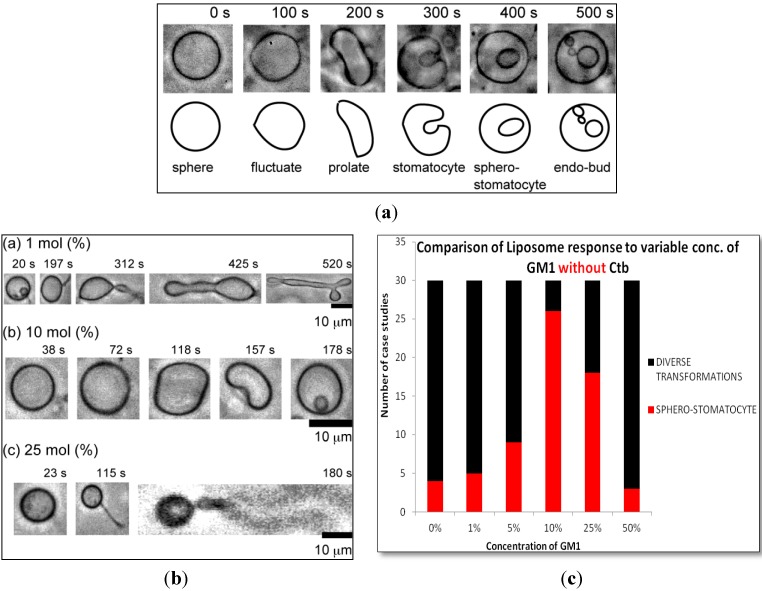
(**a**) Phase contrast microscopic image of the transformation of homogeneous DOPC liposome after addition of glucose; (**b**) Phase contrast images of the transformation of homogeneous DOPC liposomes containing (a) 1 mol %; (b) 10 mol %; (c) 25 mol % monosialotetrahexosylganglioside (GM1) (molar ratio) after the addition of glucose; (**c**) Variable concentration of GM1 without cholera toxin B subunit (Ctb).

Figure 1a shows examples of the effect of environmental stimuli on spherical single phase DOPC liposomes. As shown in figure 1a the spherical liposome begins to undulate and assume an ellipsoid shape due to osmosis. It is well known that the decrease in aqueous volume due to the osmotic pressure results in a transformation from spherical to various asymmetric shapes [10]. Thus, in the case of homogeneous liposomes, the external stimuli led to large deformations of the entire membrane surface.

#### 2.1.1. Homogeneous Liposome with Variable Concentration of GM1

Interestingly GM1 incorporation exerted an effect on GUVs in a particular manner. In Figure 1b a typical image of GUV containing 1 mol %, 10 mol % & 25 mol % molar ratio GM1 after introduction to osmotic stress is shown. Over a wide range of GM1 concentration we observed that there was a formation of diverse structures but at a certain concentration of 10 mol % GM1, we observed majorly the formation of a stable sphero stomatocyte. The formation of small sphero-stomatocyte was observed in very few GUVs. Further addition of GM1 increased the tendency of GUVs to form small sphero-stomatocyte. Figure 1c shows the statistical analysis of the overall transformations at various concentration levels of GM1.

#### 2.1.2. Homogeneous Liposome with Variable Concentration of GM1 with Ctb

**Figure 2:**
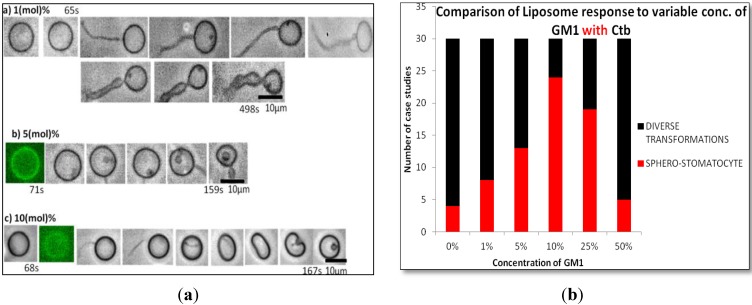
(**a**) Phase contrast images of the transformation of homogeneous DOPC liposomes containing (a) 1 mol %; (b) 5 mol %; (c) 10 mol %, GM1 (molar ratio) and Ctb (25 µg/mL) after the addition of glucose; (**b**) Variable concentration of GM1 without Ctb.

The addition of GM1 and protein (Ctb-25 mg/mL) to the liposomes at different concentration of GM1 showed similar results to that of liposome containing GM1 only. As the concentration of GM1 increased to 10%, the transformation of lipsomes to a more stable state was observed. At higher concentrations there exist more diverse structures indicating that GM1 induces stability and decreases diversity at certain concentration (Figure 2). At 10 mol % GM1, formation of a stable sphero-stomatocyte was observed. However, it was also observed that the addition of Ctb to GM1 incorporated liposome decreased the time required to attain a certain stable transformation beyond which there was not much change in the transformation of liposomes. Hence on addition of Ctb, the above diversity was unaffected but stability increased.

### 2.2. Heterogeneous Liposome

We added pure water to GUVs, thus generating hypotonic conditions and causing osmotic stress. This osmotic pressure caused swelling of the GUVs that increased the lateral tension of the membranes [11]. At 10 mol % molar concentration of GM1, we observed the transformations for three types of heterogeneous liposomes:

a. 5:5 (DOPC:DPPC) So/Ld phase;
b. 4:4:2 (DOPC:DPPC:Cholesterol) Lo/Ld phase;
c. 2:5:3 (DOPC:DPPC:Cholesterol) Lo/Ld phase.

**Figure 3:**
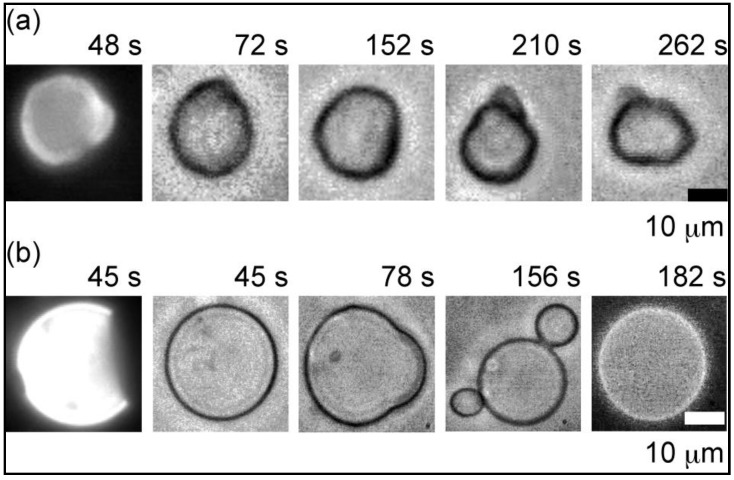
Images of the transformation of heterogeneous liposomes containing 10% GM1 after the addition of glucose. (**a**) DOPC/DPPC/Cholesterol 5:5:0; (**b**) DOPC/DPPC/Cholesterol 4:4:2.

For So/Ld phase liposome, it was observed that there was no separation of phases under osmotic stress, but continuous fluctuations are observed in such types of liposomes. Figure 3 clearly depicts an example of such a type of liposome. Also for Lo/Ld phase liposome, it was observed that, on exposure to osmotic stress, a separation of both phases takes place.

#### 2.2.1. Heterogeneous Liposome with Variable Concentration of GM1

**Figure 4:**
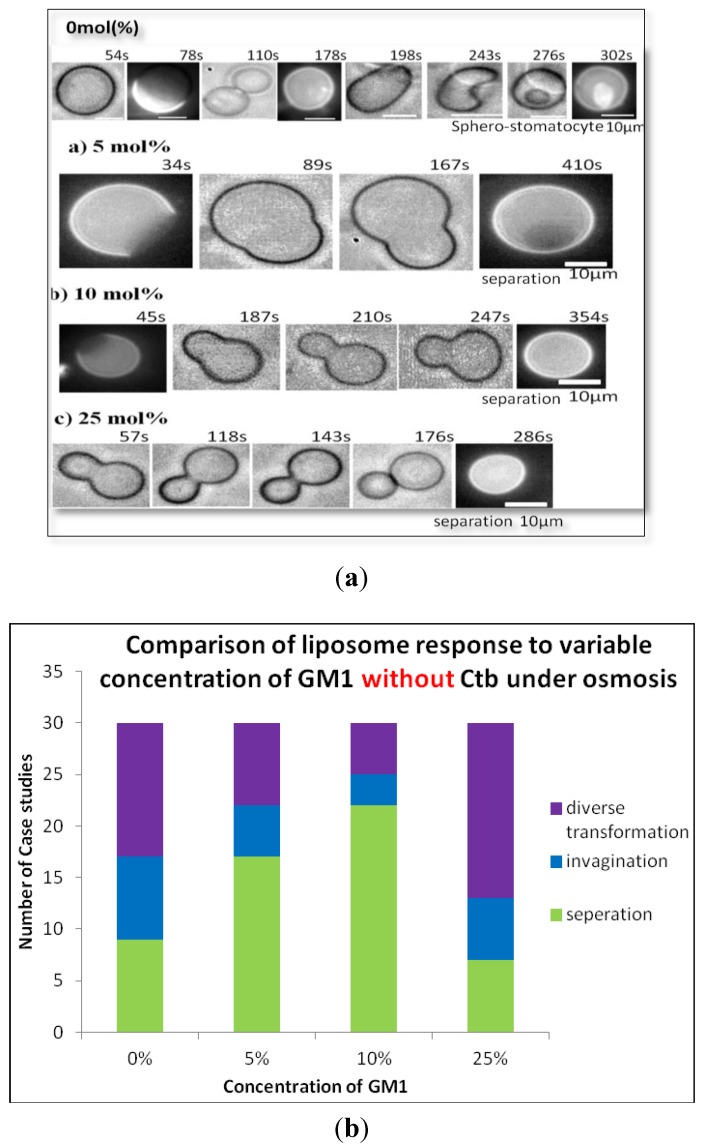
(**a**) Phase contrast images of the transformation of heterogeneous DOPC liposomes containing 0 mol % (a) 5 mol %; (b) 10 mol %; (c) 25 mol % GM1 (molar ratio) after the addition of glucose; (**b**) Variable concentration of GM1 without Ctb.

Raft membranes were incorporated with GM1. The addition of GM1 to heterogeneous liposome showed the formation of a stable transformation even at very low concentrations of GM1 (1%, 5%). As the concentration of GM1 increased, the two phases of the liposome separated, hence leading to stability. Here we also observed the separation of the two phases in the liposomes was at a maximum at 10% concentration of GM1 as shown in Figure 4. This result can easily be seen from the statistical data in Figure 4b which shows that at 10% concentration of GM1 in raft membrane, the liposome tends to exhibit raft region and forms small vesicles. Interestingly, a decrease in stability was observed at higher concentration of GM1.

#### 2.2.2. Heterogeneous Liposome with GM1-Ctb Interaction

**Figure 5:**
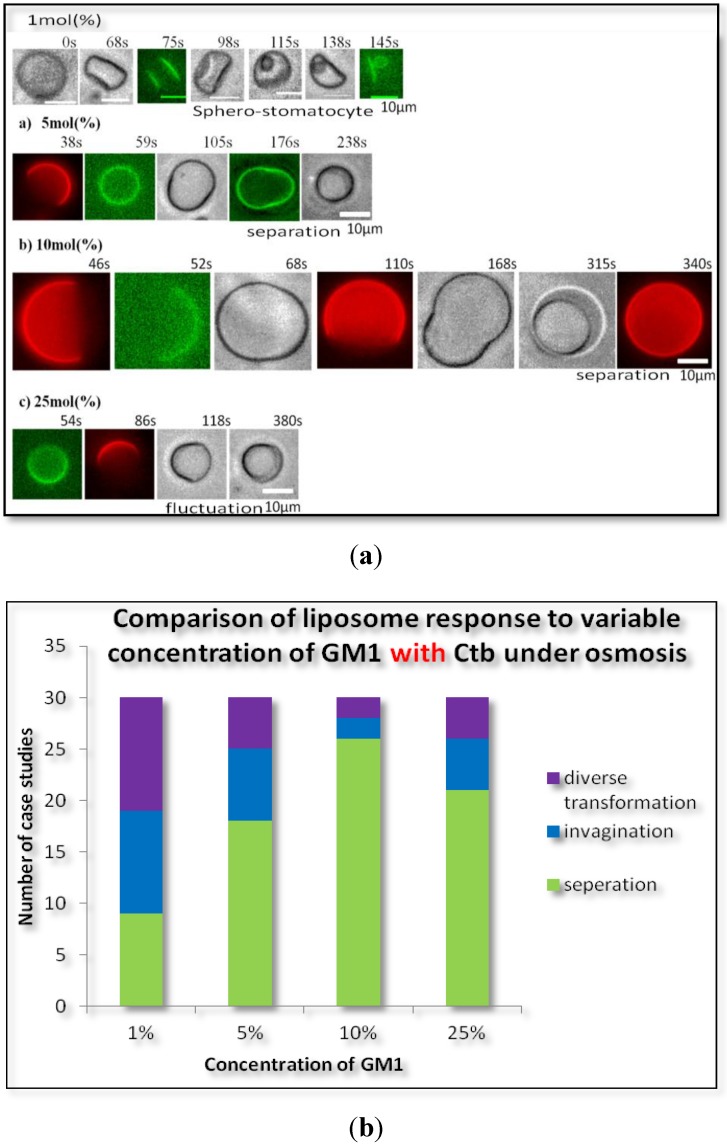
(**a**) Phase contrast images of the transformation of heterogeneous DOPC liposomes containing 1 mol % (a) 5 mol %; (b) 10 mol %; (c) 25 mol % GM1 (molar ratio) and Ctb after the addition of glucose; (**b**) Variable concentration of GM1 with Ctb.

GM1 incorporated raft membrane were introduced to CTB. GM1-Ctb interaction in heterogeneous liposome did not show much change in diversity but a noticeable change was observed in the stability *i.e*., on addition of Ctb the time required to attain a stable transformation in the liposome decreased. The various transformations in heterogeneous liposome containing variable concentrations of GM1 with Ctb and their statistical data are shown in Figure 5. Thish shows that the presence of Ctb along with GM1 on raft membrane surfaces induces such interactions that lead to fast attainment of stability.

## 3. Experimental Section

### 3.1. Materials

Unsaturated phospholipids, Dooley l-α phosphatidylcholine (DOPC), a saturated phospholipids, dipalmitoylL-phosphatidylcholine (DPPC) and cholesterol were purchased from Avanti Polar Lipids. Bovine brain ganglioside GM1 ammonium salt was purchased from Calbiochem. Fluorescent dyes, *N*-(rhodamine red-X)-1,2-dihexadecanoyl-*sn*-glycero-3-phosphoethanolamine triethylammonium salt (rhodamine red-X DHPE) (λ_ex_ = 560 nm, λ_em_ = 580 nm) and Alexa Fluor 488 conjugate cholera toxin subunit B (Ctb-488) (λ_ex_ = 495 nm, λ_em_ = 519 nm) were obtained from Invitrogen. All other reagents were purchased from Nacalai Tesque and were of analytical grade. Deionized water obtained from a Millipore Milli Q purification system was used to prepare buffers and reagents.

### 3.2. Preparation of Giant Unilamelar Vesicles (GUV)

Giant liposomes were prepared using the natural swelling method from a dry lipid film [12]. The lipid mixture dissolved in 1:2(v/v) chloroform/methanol along with rhodamine red-XDHPE in a glass test tube were dried under vacuum for 3 h to form thin lipid films [13]. Next, the films were hydrated with deionised water at 37 °C for an hour before being combined with a Ctb-488 solution. Then, the liposome was incubated for more than several hours at room temperature (24 °C). Finally D(+) glucose was added just before microscopic observations. The final concentrations were 0.2Mm lipids (DOPC/DPPC/Cholesterol = 4:4:2, 2:5:3, 5:5) with 10% GM1 and 0.2% rhodamine red-X DHPE and Ctb-488. Additionally, homogeneous liposomes without a raft phase were prepared using the same swelling method.

### 3.3. Microscopic Observations

Fluorescence images of GUV’s were acquired using an inverted fluorescent microscope (Olympus BX-51, Olympus, Tokyo, Japan) at room temperature (24 °C). Immediately before the confocal microscopy experiment, the GUV suspension was diluted with an equal volume of mixture of 2 mM glucose. This concentration difference induced osmotic stress. Images were collected at 30 frames per second and stored on the computer hard disk for further analysis.

### 3.4. Induction of Osmotic Stress

Lipid vesicles and 2 mM glucose solution were poured into a test tube and gently mixed by tapping. Observation of the vesicular dynamics was within 2 min of glucose solution introduction to the lipid vesicles at room temperature (24 °C).

## 4. Discussion

**Figure 6:**
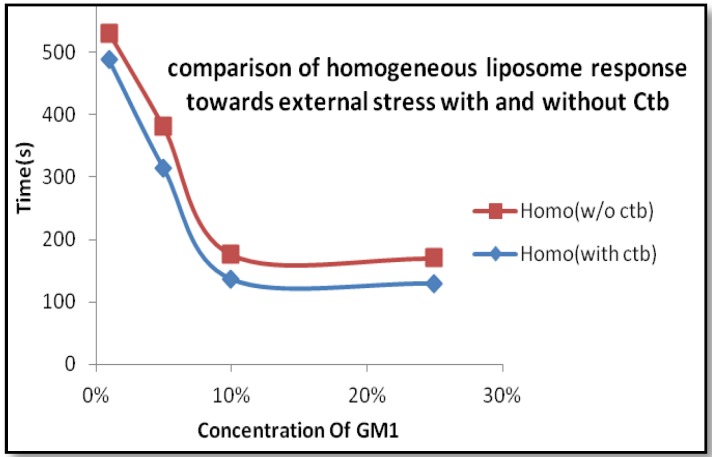
Time span comparison of Homogeneous liposome with and without Ctb.

**Figure 7:**
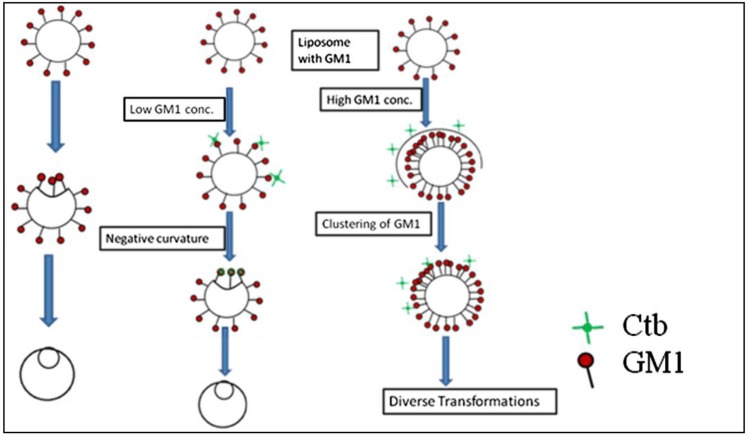
Schematic representation of the mechanism of curvature induction in the presence of GM1 and Ctb.

From the previous findings, we know that endocytic transformations in raft model membranes after the application of external stimuli demonstrate that there are two distinct pathways for the internalization process, simple budding and wavy budding [14]. The transformation in response to the stimuli can be attributed to an increase in the excess surface area. When the liposome is subjected to high osmolarity, the water efflux across the membrane reduces the inner aqueous volume [15]. The resulting aqueous membrane allows transformations in a variety of vesicular morphologies. Therefore the lateral tension induced by osmotic stress results in an increase of line tension at the Lo/Ld phase boundary which is sufficient enough to produce the lateral tension, exceeding the rupture limits of the membrane [16]. Similar research results were reported previously. In the case of homogeneous liposomes with GM1, there is a particular concentration where there exists a possibility of formation of sphero-stomatocyte morphology. In addition to this, in the case of heterogeneous liposome the domains separated in all the cases except for where there were very small domains, *i.e*., the domain size was not observed to be a major factor affecting the morphological changes. In homogeneous liposomes with ganglioside GM1, we observed a similar type of dynamics. This can be attributed to the fact that the introduction of GM1 induced the formation of negative curvature, leading to the joining of the two ends of the liposome resulting in a stable sphero-stomatocyte formation. Also the addition of Ctb to the above GM1 containing liposome, resulted in the same dynamics but the time span required to achieve that stable structure decreased by the addition of Ctb indicating that interaction between the glycosyl chain and the Ctb modifies the surface interaction processes and leads to easier and earlier morphological changes of the liposomes (Figure 6 and Figure 7).

**Figure 8:**
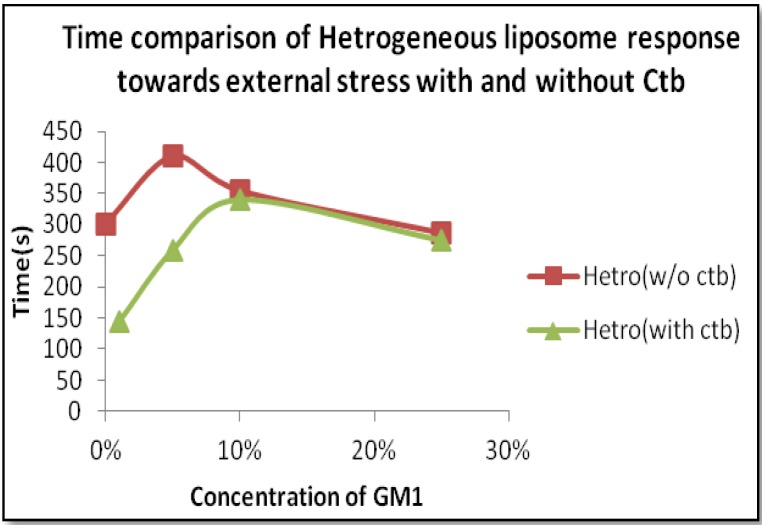
Time span comparison of Heterogeneous liposome with and without Ctb.

**Figure 9:**
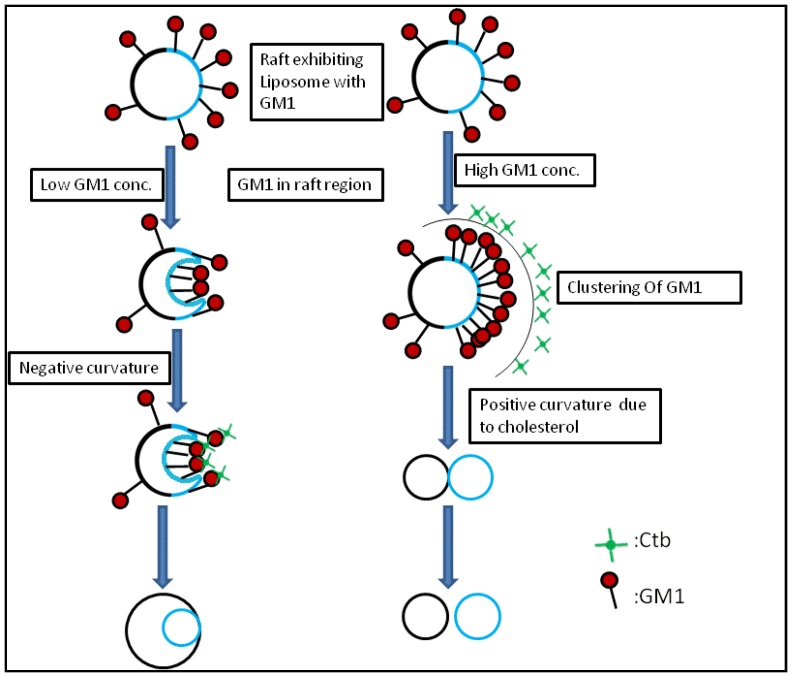
Schematic representation of the mechanism of curvature induction in the presence of GM1 and Ctb.

Considering the case of heterogeneous liposome, in the presence of ganglioside GM1 and GM1-Ctb interactions, we observed different types of dynamics. At lower concentrations of GM1, we observed the formation of a stable sphero stomatocyte whereas as the concentration of GM1 increased the formation of an eight-like structure followed by the final separation of the two phases of the liposome was majorly observed indicating that GM1 and Ctb reside in the raft region leading to the formation of a positive curvature. The formation of curvature decreased after a certain concentration of GM1 (beyond 10%) due to the clustering of GM1 molecules at higher concentration. On addition of Ctb, the same dynamics was observed but the time required in attaining the stable transformation decreased (Figure 8 and Figure 9). It is believed that the ligand receptor interaction involves attachment of the toxin to the ganglioside pentasaccaride unit via hydrogen bonds which can also form between GM1 head groups. Such pre-clustering of GM1limits its availability of attachment to the invading protein toxin, as a consequence simply increasing the receptor concentration is not sufficient.

Recently it was reported for the erythrocytes that GM1 *per se* shows low membrane curvature dependent distribution and therefore holds flexible spontaneous curvature. In contrast, the cross-linked GM1-ligand complex has a strong preference for highly outward curved membrane and possesses overall positive spontaneous curvature [17]. Furthermore, molecular dynamic simulations suggested that there appeared to be numerous hydrogen bonds among glycan portions of the GM1 clusters due to condensation [18]. Such kinds of clusters might provide a hydrophobic environment which mediates the formation of toxic amyloid β fibrils [19,20]. These recent reports may be related to our results using model membrane systems. However, more physicochemical studies are needed to show detailed physical insights of our results.

We studied the influence of ganglioside GM1 on the properties of Lo domains in GUVs. It was found that for homogeneous liposome, at 10 mol % molar ratio concentration of GM1 the formation of small sphero-stomatocyte is most favored and can be attributed as the critical cut-off concentration for the transformation. For heterogeneous liposome it was observed that at 10 mol % concentration of GM1 the tendency of the domains to separate from GUVs was the same for different compositions of liposomes.
